# Solvothermal synthesis and thermoelectric properties of indium telluride nanostring-cluster hierarchical structures

**DOI:** 10.1186/1556-276X-6-329

**Published:** 2011-04-13

**Authors:** Guo'an Tai, Chunyang Miao, Yubo Wang, Yunrui Bai, Haiqian Zhang, Wanlin Guo

**Affiliations:** 1 Institute of Nanoscience, Nanjing University of Aeronautics and Astronautics, 29 Yudao Street, Nanjing 210016, People's Republic of China; 2 College of Materials Science and Technology, Nanjing University of Aeronautics and Astronautics, 29 Yudao Street, Nanjing 210016, People's Republic of China

## Abstract

A simple solvothermal approach has been developed to successfully synthesize n-type α-In_2_Te_3 _thermoelectric nanomaterials. The nanostring-cluster hierarchical structures were prepared using In(NO_3_)_3 _and Na_2_TeO_3 _as the reactants in a mixed solvent of ethylenediamine and ethylene glycol at 200°C for 24 h. A diffusion-limited reaction mechanism was proposed to explain the formation of the hierarchical structures. The Seebeck coefficient of the bulk pellet pressed by the obtained samples exhibits 43% enhancement over that of the corresponding thin film at room temperature. The electrical conductivity of the bulk pellet is one to four orders of magnitude higher than that of the corresponding thin film or p-type bulk sample. The synthetic route can be applied to obtain other low-dimensional semiconducting telluride nanostructures.

PACS: 65.80.-g, 68.35.bg, 68.35.bt

## Introduction

Thermoelectric materials that convert electrical energy into thermal energy or vice verse have been used in cooling, heating, generating power, and recovering waste heat [1-4]. The thermoelectric performance of a given material is characterized by its dimensionless figure of merit [5]:

where σ, *S*, *k*, and *T *are the electrical conductivity, Seebeck coefficient, thermal conductivity, and absolute temperature, respectively. The thermal conductivity comprises the combination of heat carried by phonons or lattice vibrations (*k*_l_), and electrical carriers (*k*_e_). A good thermoelectric material should be perfective combination of high power factor (*S*^2^σ) with low thermal conductivity. In a bulk material, the Weidmann-Franz law limits the ratio σ/*k*, which makes optimization of *ZT *very difficult [5,6]. Recently, nanostructures lowering *k *and the distortion of electronic density of states enhancing *S *are effective approaches to enlarge *ZT *[7-10]. The *ZT *value of silicon nanowires is over 100-fold improvement up to *ZT = *0.6 to 1 than that of bulk Si at near room temperature [11,12]. A maximum *ZT *value of approx. 2.4 was observed in a p-type Bi_2_Te_3_/Sb_2_Te_3 _superlattice thin film [13] and a *ZT *value of 1.6 was also reported in PbSeTe/PbTe quantum dots [14]. Particularly, layered semiconductors such as bismuth telluride (Bi_2_Te_3_), indium selenide (In_4_Se_3_) with nanostructures are very promising for thermoelectric applications [7,13,15].

In addition, hierarchically ordered multiscale architectures have attracted great interest because of their emergent properties [16-19]. Specially, hierarchical ordered structures have great potential in developing high-efficient thermoelectric materials and devices because of very low thermal conductivity and high Seebeck coefficient [18,20]. Up to now, most hierarchical nanostructures were prepared by the surfactants or biomoleculars, which can control the shape and size of semiconductor nanomaterials but they make post-treatment for the materials very difficult and influence the optical and electrical, thermal, magnetic properties of the products [21]. Therefore, it is necessary to develop a facile, surfactant-free, and high-efficient approach to produce hierarchically structural thermoelectric materials at mild temperature and pressure.

Layered binary chalcogenide alloys A_2_^III^B_3_^VI ^(A = Al, Ga, In and B = S, Se, Te) with semiconducting properties have important applications in energy conversion and information devices [15,22-24]. Among these compounds, indium telluride (In_2_Te_3_) possessing disordered structure with respect to metal atom is a promising candidate for thermoelectric, optoelectronic, switching and memory devices [25-28]. It exhibits two crystalline phases labeled as α and β corresponding to low and high temperature formation, respectively. α-In_2_Te_3 _has a face-centered cubic (fcc) lattice with *a *= 1.850 nm, which is approximately two times more than the lattice parameter of β-In_2_Te_3 _(*a *= 0.616 nm). The transition temperature between the two phases is about 600°C [29]. To date, α- and β-In_2_Te_3 _thin films, three-dimensional open-framework In_2_Te_3 _and it's supertetrahedral T_2 _clusters have been prepared by electrochemical atomic layer deposition, thermal evaporation, electron beam evaporation, etc. [30-33]. However, synthesis and application of hierarchically one-dimensional (1D) In_2_Te_3 _nanostructures have not yet been reported, and it is necessary to understand In_2_Te_3 _material properties in low dimensionality.

In this article, a facile, surfactant-free, and high-efficient solvothermal approach has been developed to successfully synthesize α-In_2_Te_3 _hierarchical structures using ethylenediamine (EDA) as the reducing and complexing agent. The typically well-oriented nanoplatelet in the hierarchical structures possesses an edge length of approx. 700 nm and a thickness of approx. 150 nm. The Seebeck coefficient of the bulk pellet pressed by the obtained In_2_Te_3 _samples exhibits a remarkable enhancement about 43% over that of the reported corresponding thin film at room temperature.

### Experimental

All chemicals are analytical grade products purchased from Shanghai Chemical Reagent Company and were used as received without further purification.

In a typical synthesis process, 0.3071 g (0.8 mmol) of In(NO_3_)_3 _and 0.2712 g (1.2 mmol) of Na_2_TeO_3 _were put into a Teflon-lined stainless steel autoclave of 50 mL capacity and dissolved in ethylene glycol (EG) (35.56 mL) under vigorous magnetic stirring to form a clear solution at room temperature for 1 h. Then, EDA (4.44 mL) was added into the mixed solution. The solution was stirred for 30 min again. Then the autoclave was closed and maintained at 200°C for 3, 6, 12, 18, and 24 h. After the treatment, the autoclave was cooled to room temperature naturally. The black flocculating product was collected from the solution by centrifugation, washed several times with absolute ethanol and ultrapure water with resistivity of 18 MΩ-cm, and then dried at 60°C in vacuum for 10 h; as a result, the black powders were obtained.

The structural properties of the as-prepared products were analyzed by power X-ray diffractometer, which were obtained using Bruker D8 Advance diffractometer operating at 40 kV and 40 mA (Cu Kα radiation, λ = 0.154178 nm). The morphology of the as-prepared products was analyzed by field-emission scanning electron microscopy (FE-SEM, Sirion 200, 10 kV). Transmission electron microscope (TEM) images and energy dispersive X-ray spectroscopy (EDX) were obtained at 200 kV using a JEM-2010 microscope by dropping a dilute ethanol solution of the powders onto the ultrathin carbon-coated copper grids.

**Figure 1:**
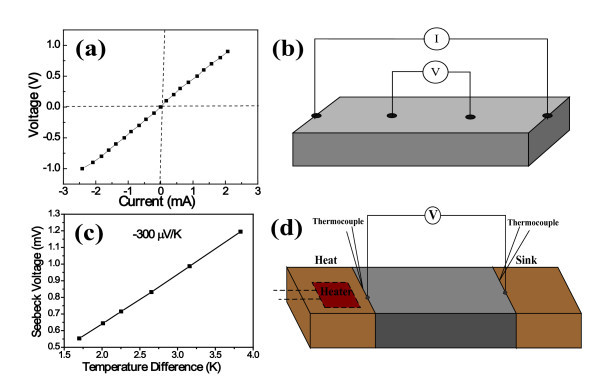
**Thermoelectric transport properties of the In2Te3 hierarchical structures**: **(a) ***I*-*V *characteristics obtained from the bulk pellet composed of the In_2_Te_3 _hierarchical structures;**(b) **The scheme of four-probe method; **(c) **Dependence of the Seebeck voltage as a function of temperature differences along the thermoelectric bulk pellet; **(d) **The set up of the measurement configuration for the Seebeck coefficient.

The obtained powders were pressed under a pressure of 58 MPa for 5 min and further pressed with a pressure of 460 MPa for 30 min at room temperature to decrease the porosity of the bulk pellet. Therefore, a rectangular bar of the powders with dimensions 15.06 mm × 5.03 mm × 1.07 mm was obtained for electrical conductivity and Seebeck coefficient measurement. A four-probe method was adopted for electrical conductivity measurement illustrated in Figure 1b. Silver pastes dropped in two ends of the thermoelectric pellet were used as electrical contacts of the electrodes to the sample. The set up of Seebeck coefficient was illustrated in Figure 1d. To decrease the effect of contact resistance on experimental results, a temperature difference of about 1 to 4 K between cool and hot ends of the bulk pellet was used for Seebeck coefficient measurement. The temperature gradient was established in the sample when the electrical power was applied by a ceramic heater. The temperature differences (Δ*T*) were determined by the nickel chromium-nickel silicon thermocouples. The two thermocouples were contacted with two ends of the pellet to determine the temperature change. Electrodes fixed on two ends of the thermoelectric pellet were used to monitor the Seebeck voltage drop between the two electrodes. Seebeck coefficients were determined from the slope of plots of sample voltage versus Δ*T*: *S *= -Δ*V*/Δ*T*.

## Results and discussion

### Phase and morphology characterization of the In_2_Te_3 _hierarchical structures

**Figure 2:**
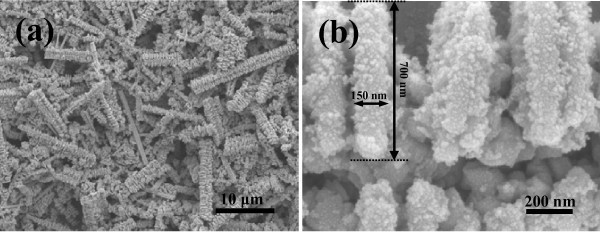
**FE-SEM images of the In**_**2**_**Te**_**3 **_**hierarchical structures prepared at 200°C for 24 h**:**(a) **low magnification and **(b) **high magnification.

**Figure 3:**
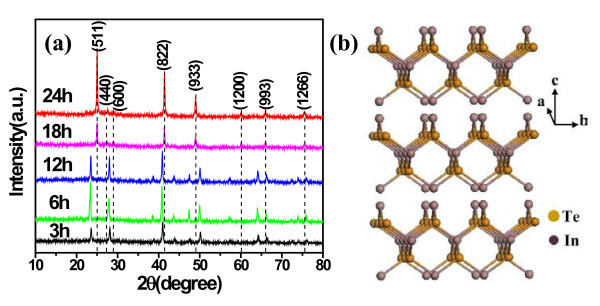
**XRD patterns and crystal structure of the as-synthesized In2Te3 hierarchical structures**: **(a) **Powder XRD patterns of the samples synthesized with different durations: 3, 6, 12, 18, and 24 h. The Miller indices correspond to bulk cubic In_2_Te_3 _crystals; **(b) **Layered crystal structure of bulk In_2_Te_3_. The peaks positions of the bulk In_2_Te_3 _XRD profile are indicated by short dashed lines for comparison.

The morphology and size of the as-synthesized products were characterized by FE-SEM on a Cu substrate. The FE-SEM images show a large number of hierarchical structures, each of which consists of arrays of small nanoplatelets which is similar to nanostring-cluster structure. Figure 2a shows a FE-SEM image of the In_2_Te_3 _hierarchical structures with diameter from 100 nm to 2 μm and length from several to over 100 μm, under a typically solvothermal reaction with In(NO_3_)_3 _and Na_2_TeO_3 _as the reactants, and EDA as the reductant and complexing agent at 200°C for 24 h. Figure 2b shows that a typically well-oriented nanoplatelet in the hierarchical structures possesses an edge length of approx. 700 nm and a thickness of approx. 150 nm. The phase purity and crystallographic structure of the products were determined by X-ray powder diffraction (XRD) with Cu Kα radiation. Figure 3a shows the transformation from *t*-Te nanowires to In_2_Te_3 _hierarchical structures occurred with increasing the reaction duration. As shown in Figure 3a, the as-synthesized In_2_Te_3 _hierarchical structures at 200°C for 24 h exhibited three broad peaks at 2θ = 25.02, 41.40, and 49.02, which were assignable to diffractions of the (511), (822), and (933) planes, respectively. In addition, five weak peaks at 2θ = 27.58, 28.92, 59.92, 66.02, and 75.46 were assignable to diffractions of the (440), (600), (1200), (993), and (1266), respectively. The diffraction peaks can be indexed to the purely fcc phase of In_2_Te_3 _(space group: F4\overline 3 m, no. 216) with lattice constants of *a *= *b *= *c *= 1.848 nm (JCPDS card: 33-1488). No other peaks from any other phases of indium telluride were detected. The crystal structure, as seen in Figure 3b, shows strong covalent bonding within each layer and a weak van der Waals force between the layers. Thus, the appearance of nanoplatelets on the wires is understandable due to the planar sheetlike nature of the building blocks. In other words, growth of the nanoplatelets is actually the outward embodiment of the internal crystal structure in this case.

**Figure 4:**
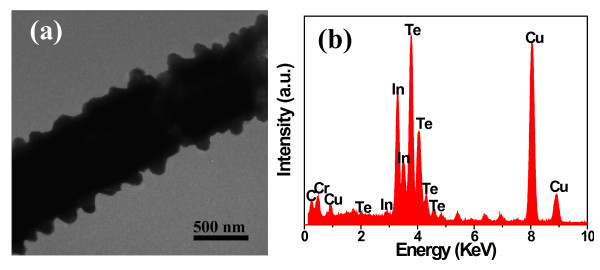
**TEM image and EDX pattern of the as-synthesized In2Te3 hierarchical structures**: **(a) **TEM image of the In_2_Te_3 _hierarchical structures prepared at 200°C for 24 h; **(b) **EDX pattern of the samples taken from the TEM image. The signals for Cu, Cr peaks are originated from the substrate.

The detailed structure of the products was further characterized by TEM. Figure 4a exhibits the TEM image of a typical α-In_2_Te_3 _hierarchical structure. Reliable electron diffraction patterns and high-resolution TEM images are difficult to obtain because of the partial melting of the samples under electron-beam irradiation at 200 kV, which could be obviously seen in Figure 4a. However, XRD pattern (Figure 3a) and FE-SEM images (Figure 2) show that the obtained hierarchical structures are polycrystalline. EDX spectrum reveals an atomic ratio of In:Te = 42.81:57.19 (Figure 4b), close to the stoichiometric In_2_Te_3 _within experimental errors. The signals for Cu, Cr peak were originated from the substrate.

### Growth mechanism of the In_2_Te_3 _hierarchical structures

**Figure 5:**
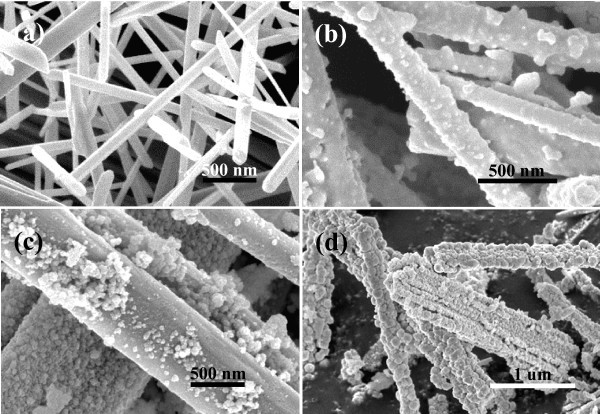
**FE-SEM images of the as-synthesized samples at different time intervals**: **(a) **3 h, **(b) **6 h, **(c) **12 h, and **(d) **18 h.

**Figure 6:**
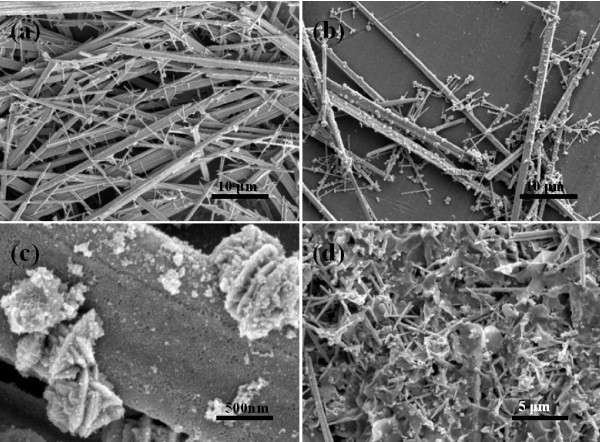
**FE-SEM images of the as-synthesized samples at different temperatures and durations**: **(a) **160°C for 24 h, **(b) **and **(c) **160°C for 48 h, and **(d) **220°C for 24 h.

**Figure 7:**
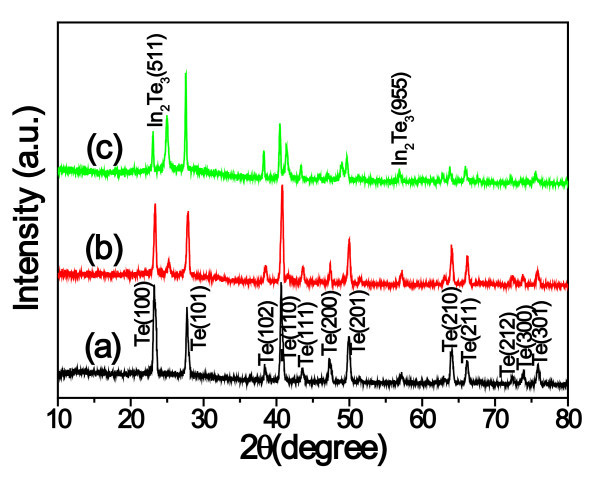
**Powder X-ray diffraction patterns of the as-synthesized samples with different temperatures and durations**: **(a) **160°C for 24 h, **(b) **160°C for 48 h, and **(c) **220°C for 24 h.

To understand the growth process of the In_2_Te_3 _hierarchical structures, the effects of the reaction duration, temperature, volume ratio of EDA/EG, concentration of In(NO_3_)_3 _on the resulting products were systematically investigated (Figure 3, 4, 5, 6 and 7). (1) Time-dependent experiments in EDA at 200°C were performed to gain insight into the formation process of the hierarchical structures. The obtained product was nearly pure hexagonal *t*-Te nanowires within 3 h at 200°C (Figures 3a and 5a). Some flower-like In_2_Te_3 _nanoparticles appeared on the surface of the nanowires when the reaction duration was increased to 6 h (Figure 5b). Prolonging the reaction duration to 12 h, the same results as that of 6 h were produced (Figures 3a and 5c). Extending the reaction duration to 18 h caused smooth nanowires to grow into the wires with rough surface composed of nanoparticles (Figure 5d). The above results show that the intermediate morphology composed of the Te nanowires and In_2_Te_3 _nanoplates can be produced in 6 and 12 h. Well-defined In_2_Te_3 _hierarchical structures were obtained when prolonging the reaction duration up to 24 h (Figures 2 and 3a). Besides the ordered nanoplates, some nanowires were detected in the nanostructure, which suggests that the nanoplates nucleate and grow out of the nanowires. (2) When aged at 160°C over 48 h, the nearly pure hexagonal *t*-Te nanowires were only formed (Figures 6b and 7b), but the FE-SEM images (Figure 6c) shows that some flower-like In_2_Te_3 _nanoparticles appeared on the surface of the nanowires. When aged at 220°C over 24 h, the mixture of nanowires and nanoplates was produced, as shown in Figure 6d. Figure 7c exhibits the mixture mainly composed of *t*-Te nanowires. The indexed Te peaks are in good agreement with the standard literature data (JCPDF card number: 36-1452) in Figure 7. (3) The volume ratio of EDA/EG also plays an important role in structure and phase control of the resulting products (not shown). When pure EDA or EG was used in the reaction, pure cubic In_2_Te_3 _could not be got (not shown). Additionally, when the volume ratios of EDA/EG were 1:2, 1:12, or 1:16, pure cubic In_2_Te_3 _could also not be obtained. (4) When the other conditions remained the same, the concentration of In(NO_3_)_3 _was 13.25 mM, the obtained products were a mixture of In_2_Te_3 _and *t*-Te (not shown).

On the basis of the above experimental results, a diffusion-limited reaction mechanism can be reasonably used to explain the formation of the In_2_Te_3 _hierarchical structures. The chemical reactions to form the hierarchical structures are formulated as follows:(1)(2)(3)(4)(5)

**Figure 8:**
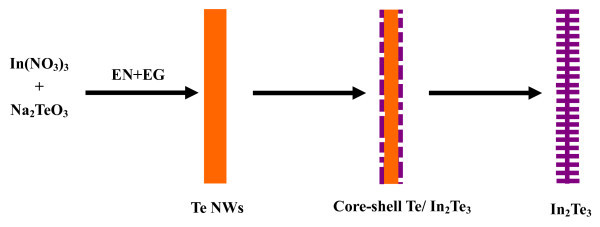
**A schematic of the formation mechanism of the In**_**2**_**Te**_**3 **_**hierarchical structures**.

In the reaction system, acetaldehyde is produced by dehydration of EG at high temperature, as shown in Equation 1. The EG acted as both solvent and reductant in this case, and  ions were reduced by EG to form elemental Te at the beginning of the reaction, which is shown in Equation 2. The reaction (2) was a quick step, which determined the formation of *t*-Te nanowires at the beginning of the reaction. The EDA plays an important role in the transformation process from the *t*-Te nanowires to the hierarchical In_2_Te_3 _nanowires because strongly basic solvents, such as ammonia and EDA, can reduce Te into Te^2- ^with a low speed (Equation 3). This leads to the formation of nanoporous structures on the surface of the nanowires (Figure 5b, c); meanwhile, a complex is formed by the reaction of the solvent EDA molecule and the metal In^3+ ^ions. In this complex, each In^3+ ^is surrounded by six NH_2 _group, similar to Co^3+ ^[34]. Although In^3+ ^is combined with the *t*-Te and reduced into indium by the EDA *in situ *near the surface of the template *t*-Te nanowires at high temperature and high pressure, as Equation 4, In^3+ ^has little solubility in EDA at room temperature and atmosphere [35]. Thus, indium atoms can quickly diffuse into the *t*-Te nanowires. With increasing the reaction duration, the molar ratio of In:Te is close to the stoichiometric composition of In_2_Te_3 _(Figure 4b). The transformation mechanism of the *t*-Te nanowires to the In_2_Te_3 _hierarchical structures is shown in Figure 8.

### Thermoelectric transport properties of the In_2_Te_3 _hierarchical structures

To evaluate thermoelectric properties of the In_2_Te_3 _hierarchical structures, the room-temperature electrical conductivity and Seebeck coefficient of the bulk pellets were measured, which were pressed using the as-prepared powders at 460 MPa for 30 min. The Seebeck voltage measured between cool and hot ends of the bulk pellet varies linearly with the temperature difference. The slope yields a Seebeck coefficient of -300 μV/K (Figure 1c). The negative sign indicates that the bulk pellet behaves as a n-type semiconductor, which is consistent with the EDX result (Figure 4b). The Seebeck coefficient of the bulk pellet is about 43% enhancement over 210 μV/K of the reported corresponding best thin film sample (seen in ref. [25]). Additionally, the bulk pellet exhibits a linear current-voltage (*I-V*) curve (see Figure 1a) that is symmetric about the origin, indicating that the contacts are ohmic. The slope yields a resistance of 436 Ω. An electrical conductivity σ of 6.42 Ω^-1 ^m^-1 ^can be obtained by using an average bulk pellet thickness in a 1D electrical transport model, which is one to two orders of magnitude higher than that of the corresponding thin film sample (0.66 Ω^-1 ^m^-1^) [26,36], and four orders of magnitude higher than that of the corresponding p-type bulk sample [37]. In addition, bulk In_2_Te_3 _has very small thermal conductivity about 1.4 W (m K)^-1 ^[25]. The thermal conductivity will further decrease by nanostructuring corresponding thermoelectric materials [2,38].

## Conclusions

In summary, a simple, reproducible, surfactant-free, and high-efficient solvothermal approach has been for the first time developed to successfully synthesize n-type α-In_2_Te_3 _thermoelectric nanomaterials. The nanostring-cluster hierarchical structure were prepared using In(NO_3_)_3 _and Na_2_TeO_3 _as the reactants, EDA as the reducing and complexing agent, and EG as the reductant and solvent at 200°C for 24 h. The typically well-oriented platelet in the hierarchical structures possesses an edge length of approx. 700 nm and a thickness of approx. 150 nm. A diffusion-limited reaction mechanism based on the XRD patterns and FE-SEM images with different durations was proposed to explain the formation of the In_2_Te_3 _hierarchical structures. *t*-Te nanowires are formed initially using EG as the reductant. Then In^3+ ^is reduced into indium by EDA at high temperature and high pressure. Finally, the hierarchically structural In_2_Te_3 _can be obtained by reacting indium and *t*-Te. The room temperature Seebeck coefficient of the bulk pellet pressed by the obtained samples exhibits a 43% enhancement over that of the reported corresponding thin film. The electrical conductivity of the bulk pellet is one to four orders of magnitude higher than that of the corresponding thin film or p-type bulk sample. This is a promising approach to grow semiconducting telluride nanostructures through a solution-based chemical route under controlled conditions without the presence of any catalysts or templates.

## Abbreviations

EDA: ethylenediamine; EDX: energy dispersive X-ray spectroscopy; EG: ethyleneglycol; FE-SEM: field-emission scanning electron microscopy; TEM: transmission electron microscope; XRD: X-ray powder diffraction.

## Competing interests

The authors declare that they have no competing interests.

## Authors' contributions

The work presented here was carried out in collaboration between all authors. GT designed and guided all aspects of the work. CM, YW, and YB carried out the experiments. HZ, and WG participated in the design of the study and revised the manuscript. All authors have contributed to, seen, and approved the manuscript.
